# The Glioblastoma Microenvironment: Morphology, Metabolism, and Molecular Signature of Glial Dynamics to Discover Metabolic Rewiring Sequence

**DOI:** 10.3390/ijms22073301

**Published:** 2021-03-24

**Authors:** Assunta Virtuoso, Roberto Giovannoni, Ciro De Luca, Francesca Gargano, Michele Cerasuolo, Nicola Maggio, Marialuisa Lavitrano, Michele Papa

**Affiliations:** 1 Laboratory of Neuronal Networks, Department of Mental and Physical Health and Preventive Medicine, University of Campania ‘‘Luigi Vanvitelli”, 80138 Naples, Italy; assunta.virtuoso@unicampania.it (A.V.); f.gargano@unicampus.it (F.G.); michele.cerasuolo97@gmail.com (M.C.); michele.papa@unicampania.it (M.P.); 2 School of Medicine and Surgery, University of Milano-Bicocca, 20900 Monza, Italy; marialuisa.lavitrano@unimib.it; 3 Department of Biology, University of Pisa, 56126 Pisa, Italy; roberto.giovannoni@unipi.it; 4 Department of Neurology, Sackler Faculty of Medicine, Sagol School of Neuroscience, Tel Aviv University, Tel Aviv 6997801, Israel; nicola.maggio@sheba.health.gov.il; 5 Department of Neurology, The Chaim Sheba Medical Center, Ramat Gan 5211401, Israel; 6 SYSBIO Centre of Systems Biology ISBE-IT, University of Milano-Bicocca, 20126 Milan, Italy

**Keywords:** microglia, astrocytes, high-grade glioma, cross-talk, hypoxia, metabolism, oxidative phosphorylation, glycolysis, modules, disease progression

## Abstract

Different functional states determine glioblastoma (GBM) heterogeneity. Brain cancer cells coexist with the glial cells in a functional syncytium based on a continuous metabolic rewiring. However, standard glioma therapies do not account for the effects of the glial cells within the tumor microenvironment. This may be a possible reason for the lack of improvements in patients with high-grade gliomas therapies. Cell metabolism and bioenergetic fitness depend on the availability of nutrients and interactions in the microenvironment. It is strictly related to the cell location in the tumor mass, proximity to blood vessels, biochemical gradients, and tumor evolution, underlying the influence of the context and the timeline in anti-tumor therapeutic approaches. Besides the cancer metabolic strategies, here we review the modifications found in the GBM-associated glia, focusing on morphological, molecular, and metabolic features. We propose to analyze the GBM metabolic rewiring processes from a systems biology perspective. We aim at defining the crosstalk between GBM and the glial cells as modules. The complex networking may be expressed by metabolic modules corresponding to the GBM growth and spreading phases. Variation in the oxidative phosphorylation (OXPHOS) rate and regulation appears to be the most important part of the metabolic and functional heterogeneity, correlating with glycolysis and response to hypoxia. Integrated metabolic modules along with molecular and morphological features could allow the identification of key factors for controlling the GBM-stroma metabolism in multi-targeted, time-dependent therapies.

## 1. Introduction

Glioblastoma (GBM) classified as a grade IV astrocytoma is one of the most aggressive tumors to treat [1]. The current treatment consists of surgery followed by radiotherapy and adjuvant chemotherapy with temozolomide [2]. The removal of the tumor bulk can be challenging due to localization (difficult surgical access, adjacent spared eloquent regions) and size with many subtypes of GBM found to be radio/chemo-resistant [3,4]. Moreover, GBM shows a very high cellular, epigenetic, and genetic heterogeneity, making therapeutic approaches difficult [5]. Eventually, GBM infiltrates brain tissue distant from the initial tumor mass, which frequently could not be identified because of the high migratory potential of GBM’s cells.

Recent studies elucidated the mechanisms of the crosstalk between the brain tumor and its environment since the GBM has never been described in extra-cranial sites. Neoplastic cells in the brain interact with the resident cells (neurons, astrocytes, microglia, oligodendrocytes) entangled in the extracellular matrix (ECM) and the vasculature, altering the neurovascular unit [6]. GBM is electrically and synaptically integrated into neural circuits [7,8] and glial cells play a prominent role in the progression of cancer, while neuronal progenitors’ dysfunction or de-differentiated mature cells may be involved in glioma genesis [9,10,11,12]. The single-cell RNA sequencing on glioma stem cells from human tumors revealed a transcriptional gradient over two cellular states, which have normal neural development and inflammatory wound response features [13]. The new perspective in the research on GBM is the combination of multiple approaches directed to the primary tumor and the neighboring tissue. The study of the microenvironment in the peritumoral tissue is an appealing target to sensibilize cancer cells to current and future therapies. The intercellular communication between glioma and the brain mainly occurs through nanovesicles or non-vesicular-mediated secretion. The nanovesicles contain DNA, RNA, and proteins and are taken up by immune cells as well as astrocytes and oligodendrocytes, developing somatic and epigenetic signaling to foster tumor progression. GBM affects central nervous system (CNS) elements also by cell–cell interactions [14,15,16], involving relevant plasticity in cell morphology, functions, and the bioenergetic machinery [17]. Cellular metabolism is important for region-specific neuronal toxicity in neurodegeneration [18] prompting the question of whether the metabolic changes could be the cause or the consequence of tumoral growth. Tissue context shapes the tumor metabolic adaptations, and the most favorable are selected in a specific environment. Mutations encoding for *Epidermal Growth Factor Receptor* (*EGFR*) and the *isocitrate dehydrogenases* (*IDH1* and *IDH2)* are the most common GBM-mutated metabolic genes. Amplified *EGFR* involves pathways to control glioma glycolysis and lipogenesis, while *IDH* mutations link the metabolism rewiring to epigenetic regulation (reviewed in [19] and further described in the next section). Mitochondrial crucial involvement in cancer pathophysiology was described by Otto Warburg at the beginning of the 20th century [20]. According to Warburg’s hypothesis, tumor-associated mutations might not be sufficient to induce malignant transformation if cells’ mitochondria are healthy [21]. Mitochondria and the bioenergetic machinery became a hot spot in cancer research and several compounds are currently under investigation to modulate the GBM metabolism [22,23]. However, little is known about the metabolic differences between stromal cells in the tumor tissue and cancer cells, and how these differences could interfere with the therapeutic targeting of the metabolic pathways [24]. Recent data on the CNS structure prompted the study of tumor metabolic rewiring as the expression of the networking modular activity of the different components, and not only of the cancer cells [18,25]. Systems metabolomics data analyzed by artificial intelligence techniques could be of great implementation to this aim [26,27].

Glial cells are the first elements to communicate with GBM and their immune role is well documented. We will review here the metabolic strategies of cancer and the cellular alterations found in the glia associated with GBM, highlighting the morphological, molecular, and metabolic features. We will also examine the role of macrophages, although these cells share a different genetic profile with microglia, and probably the ontogenetic origin [28,29]. Morphological changes and the molecular expression profile can be associated with defined metabolic states, related to pathophysiological mechanisms that will offer new therapeutic strategies.

## 2. Cancer Metabolic Strategies and the Role of the Microenvironment in Glioblastoma

### 2.1. The Warburg Effect and the Reverse

Since 2016, the WHO classified two forms of GBM—(i) *IDH*-wildtype (wGBM) and (ii) *IDH*-mutant (mGBM). IDH is an enzyme that catalyzes the oxidative decarboxylation of isocitrate to produce α-ketoglutarate (α-KG) and carbon dioxide (CO_2_) in the tricarboxylic acid (TCA) cycle. The wGBM is more frequently a de novo tumor, the mGBM is a GBM developed by a lower grade glioma [30], thus supporting the GBM hypothesis as a metabolic disorder. Warburg demonstrated that “respiration” in cancer cells is impaired, even in the presence of oxygen [20]. Cancer cells selectively extract nutrients from the extracellular space and upregulate GLUT3 to promote tumorigenic properties [31] via glycolysis. This process is called “aerobic glycolysis”. However, cancer cells survive in glucose-starvation condition [32], undergoing a metabolic rewire, that allows them to proliferate, invade, and resist to therapies. Indeed, GBM cells show to be highly plastic to changes in nutritional supply [33]: the mitochondrial dysfunction (Warburg effect) is not a general feature of all the cancer cells within the tumor mass, as the existence of the oxidative cancer cells was proved. The multi-omic analysis helped to define a new classification of the GBM into four subtypes that embody metabolic and developmental features. The mitochondrial GBM subtype mainly contains oxidative cancer cells, which decrease the glycolysis and rely on oxidative phosphorylation (OXPHOS) [34,35]. This is known as “the reverse Warburg effect”. The Warburg effect and the reverse Warburg effect are adaptive mechanisms made by cells according to the phase of the cell cycle and the composition of the tumor microenvironment [36]. The Warburg effect occurs in normal cells during the early embryogenesis when the oxygen availability is low [37]. Evidence also shows the case of tumor cells that have both impaired glycolysis pathway and ATP (adenosine triphosphate) production with OXPHOS. In this case, the production of high-energy phosphate would be supported by glutaminolysis. The glutamine-derived succinate can provide adequate ATP through mitochondrial substrate-level phosphorylation (mSLP) to sustain GBM growth when OXPHOS is off [38].

### 2.2. The Functional Symbiosis

Two main niches are distinguished in the tumor: (i) the perivascular niche and (ii) the hypoxic niche. Expanding tumor cells reside in the peripheral and vascularized part of the tumor, while cancer stem cells have been found mostly in the hypoxic core [39], and show a low proliferation rate in quiescence. Mature cancer cells and the cancer stem cells seem to exist in a functional symbiosis [40,41]. Cancer cells at the perivascular niches (where the oxygen and nutrient levels are high) spare the glucose for the cancer stem cells in the hypoxic area. Hypoxia induces the hypoxia inducing factor 1 (HIF-1), which is involved in several functions, including the overexpression of the genes for glycolytic pathway, the stem-like phenotype, and the self-renewal of neural precursors [42,43,44]. These cells would accelerate glucose consumption, releasing lactate. Lactate could be converted to pyruvate and used for OXPHOS in the perivascular niche, fulfilling the ATP and biosynthesis recognized need, limiting the microenvironment acidification. Relevant to this, OXPHOS metabolism is a hallmark in the differentiated state of the tumor [45]. The hypoxic tumor cells can also use carbon skeletons from glycolysis and glutamine through a truncated TCA cycle followed by mitochondrial reductive carboxylation of αKG, which does not depend on the oxygen availability. An active truncated TCA cycle can be due to the need for the biosynthesis of precursors such as lipids or nucleic acids, as well as antioxidant glutathione synthesis, rather than oxidative metabolism [46]. The symbiotic hypothesis for GBM is supported by data showing that the mitochondrial biogenesis via cyclic adenosine monophosphate (cAMP) and the metabolic switch to OXPHOS drives the differentiation of tumor cells, while the anaerobic utilization of high energy substrates such as pyruvate and lactate is associated with the expression of genes for stemness features [47,48].

### 2.3. The Role of the Microenvironment

Cancer-induced metabolic alterations within the microenvironment play a key role in tumor maintenance or else may be involved in carcinogenesis. Interestingly, oxidative stress could be the central core of metabolic rewiring. The reactive oxygen species (ROS) diffuse from cancer cells to stromal cells, which in turn result in oxidative stress. The oxidative stress induces a metabolic shift mainly through the activation of HIF-1 and nuclear factor kappa-light-chain-enhancer of activated B cells (NF-kB). These transcription factors stimulate the angiogenesis to increase oxygen availability [49,50], and the conversion into a perivascular niche. They trigger mitochondrial dysfunction and aerobic glycolysis, autophagy, and lysosomal degradation with the release of high-energy substrates such as pyruvate and lactate. Proliferative cancer stem cells would take up these molecules and use them for OXPHOS; yet, the reverse Warburg effect occurred [51]. However, studies on human breast cancer showed that mitochondrial dysfunction and autophagy-mediated catabolism in the stromal cells serves the anabolic growth of the tumor cells with enhanced mitochondrial biogenesis and OXPHOS, not accounting for the angiogenesis [52]. GBM-associated stromal cells (GASCs) functionally remind the cancer-associated fibroblasts (CAFs) described in the stroma of carcinomas, promoting the tumor in vivo and in vitro [53]. Stromal cells such as non-neoplastic-astrocytes in contact with GBM could transfer mitochondrial DNA and mitochondria via connexin (Cx) family of protein and the gap junction channels or by subcellular transporting mechanisms such as tunneling nanotubes and microvesicles, playing a key role in the GBM progression, even greater with the extracellular matrix enriched in hyaluronic acid [54]. A recent study reported that mitochondrial transplantation, from healthy astrocytes, redirects the aerobic respiration in glioma cells, attenuates the Warburg effect, and may enhance the radio sensitivity [55]. The metabolic coupling is similar to the physiological neuron-glia relationship. Neurons are essentially oxidative cells equipped with the uptake of lactate while astrocytes and microglia show a high glycolytic rate and release lactate, accomplishing the needs of the neurons [56]. However, the metabolic differentiation profiles can be reversed by regulating the activity of pyruvate dehydrogenase [57].

## 3. GBM-Associated Microglia

### 3.1. Morphology

The innate immune system, namely microglia, and macrophages, rapidly respond to alteration of the CNS homeostasis, including brain tumors. The resident immune system sustains the inflammation, phagocytoses the exogenous agents, clears the cellular debris, promotes synaptic plasticity, tissue repair, and axonal regeneration [58], and displays morphological and functional heterogeneity across injury and regions [59]. Their role in GBM biology is controversial. The depletion of microglia impairs glioma growth and invasiveness both in organotypic slice culture and in vivo tumor models [60,61,62] while, the “natural” microglia and macrophages induce glioma cell cycle arrest and differentiation in culture [63].

In the healthy brain, professional macrophages are located around the vessels and in the proximity of the ventricular areas [64], while microglia represents 5–20% of the glial population [65]. Microglial cells show a “spider” shape. A little, triangular-shaped body and very thin extensions with several ramifications to scan the safety of the brain areas. In presence of injury or modifications in the microenvironment, danger-related signal molecules (damage-associated molecular patterns, DAMPs, and pathogen-associated molecular patterns, PAMPs) bind surface receptors on microglia, triggering the intracellular activation [66,67]. Microglial cells undergo a cytoskeletal re-organization, showing a large and rounded body (ameboid-shape), with short ramification [68]. In vivo imaging showed that microglial cells sense the glioblastoma and become activated as soon as 30 min after the tumoral cells seeding, in an orthotopic mouse model [69]. Microglia and macrophages are recruited as activated cells by GBM through the release of chemoattractant factors, mainly monocyte chemoattractant protein 1 (MCP1), colony-stimulating factor-1 (CSF-1), granulocyte-macrophage colony-stimulating factor (GM-CSF), and osteopontin [70,71]. A recent study identified a spectrum of three new tumor microglial subtypes: (i) phagocytic (ii) interacting, and (iii) mobile GBM-associate microglial cells in vivo. The phagocytic subtype had a huge soma with nearly no processes. The interacting microglia with fast-moving thick processes was located in the close surrounding microenvironment of the GBM bulk. The mobile subtype was described by amoeboid-like cell shape, traveling through the tumor tissue and homing in the perivascular space [72]. Differences in microglia morphology were described between the invasive and non-invasive margins of GBM, with invading lesions presenting a ramified morphology [73]. Microglia adopts an elongated shape, moves haptotactically, and shows the phagocytic capacity in the hypoxic region of pseudo palisades, a hallmark of GBM. These features could be related to the generation of new blood vessels and debris clearance for facilitating tumor invasion [74]. Microglia appears to increase as a result of proliferation and migration at the tumor site, and its morphological activation remains circumscribed in the tumor area [75], indicating the occurrence of direct/indirect interaction with the tumoral cells. According to our unpublished results, a study showed that they infiltrate the tumor bulk only in the last phases of the tumor progression [69]. However, the precise molecular mechanisms of interaction between microglia and GBM are not known and may be different in grey matter and white matter [29,76]. Activated microglia can extend along the corpus callosum during the glioma invasion [77], secrete several factors to degrade the extracellular matrix, and facilitate the way for the cancer migration [78], rather than performing the immunological function, such as cytotoxicity, phagocytosis, and antigen presentation [79]. The role of microglia during early glioma genesis is substantially unclear. In the early stages of glioma genesis, the innate responses of microglia can be helpful, targeting the elimination of these cells [80]. However, during the progression of the malignancy, tumor cells escape the immune editing and the activated microglia starts supporting the GBM [81]. As reported, microglia co-cultured with rat C6 glioma cells for 6 h showed increased ED-1 expression (a marker for activated microglia) and morphological activation, while lacking phagocytic activity after 24 h, although the characteristic morphological features of the activated state and the expression of ED-1 were maintained [82]. Microglia interacting with GBM showed overall downregulation of detection damage and host defense capacity after 4 weeks in a mouse model [83].

### 3.2. Molecular Profile

Among the DAMPs due to the tumor presence, the extracellular accumulation of ATP is slowly hydrolyzed by GBM [84] and promotes the expression of macrophage inflammatory protein-1alpha (MIP-1α) and MCP1 in microglia and macrophages via purinergic receptor X7 (P2RX7) in a Ca^2+^-dependent mechanism [85]. The microgliosis increases over time during GBM progression but is accompanied by a failure in the pro-inflammatory tumor necrosis factor-alpha (TNF-α) secretion [86]. These data indicate a functional alteration in the late stage of tumor progression, and potentially provide a time-window for therapeutic opportunities to prevent their functional impairment. Based on the molecular profile of cytokines and chemokines expression, in vitro analysis revealed tumor-associated microglia-macrophages (TAMs) conversion from the classical-activated M1 phenotype to the classically alternative activated M2 phenotype. M1 profile is signed by the expression of the signal transducer and activator of transcription 1 (STAT1), the pro-inflammatory cytokines interleukin-1beta (IL-1β), interleukin-2 (IL-2), interleukin-12 (IL-12), TNF-α, interferon-gamma (IFN-γ), a disintegrin and metalloproteinase (ADAM)-10, ADAM-17 and phagocytic functions, and drives the anti-tumor response. Whereas, the alternative phenotype M2 can be targeted by the signal transducer and activator of transcription 3 (STAT3), interleukin-6 (IL-6), interleukin-10 (IL-10), tumor necrosis factor-beta (TNF-β), and arginase production, matrix metalloproteinases (MMP)-9 and MMP-14, and promotes glioma progression [15,87,88,89]. M2 polarization inhibits the phagocytosis and mediates the inactivation of the cluster of differentiated (CD)8+ T lymphocytes, CD4+ T helper (Th)1, and Th17 cells while promoting the function of tumor-supportive CD4+ regulatory T cells [90]. M2 cells are further divided into M2a, M2b, and M2c subpopulations. M2a is suggested to be involved in the tumor progression and has a potential dual role in inflammation [91]. M2b polarization triggers the immune complex; M2c polarization occurs in response to specific anti-inflammatory factors such as IL-10 and glucocorticoids [92,93,94].

The in vivo molecular settings do not always match since microglia and macrophages may acquire a continuum of phenotypes between M1 and M2 [95], as well as the IL-1β (common marker of M1 profile) upregulation in intra-tumoral macrophages was shown to favor glioma growth [96]. Moreover, human glioblastoma-associated myeloid cells were aligned to an undifferentiated but active M0 molecular profile, rather than M2 [97]. The fixation of the polarization profile is not reliable, due to the plasticity and heterogeneity of these cell populations, depending on the microenvironmental stimuli, metabolic reprogramming, and epigenetic imprinting [98]. On the other hand, neuro-inflammation itself is a double-edged process. The M2 response typically follows the M1 in the healthy brain and is important for wound healing and resolving inflammation [99]. It is unclear whether immune cells acquire tumor supportive phenotypes due to physiological homeostatic mechanisms to intensify immunological reactivity or are reprogrammed by GBM to gather immune-escape and support its growth and invasiveness [81,100,101,102].

### 3.3. Metabolism

**Figure 1:**
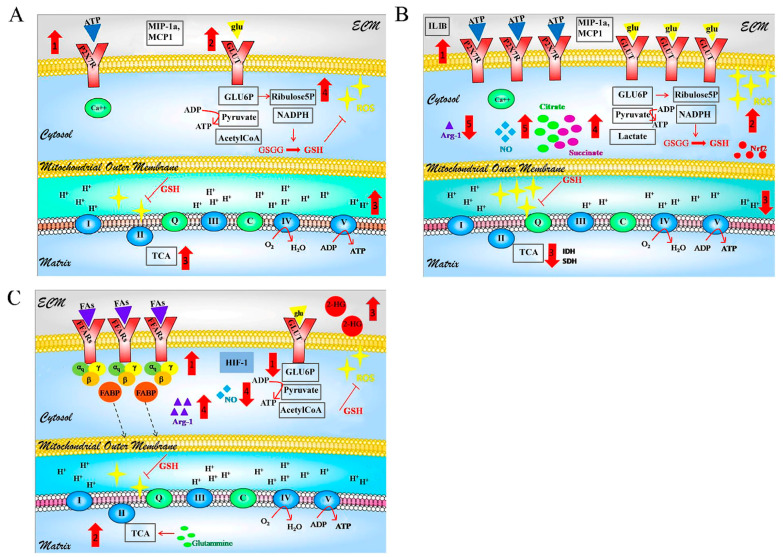
Glioblastoma (GBM)-associated reactive microglia. Activation phases scheme. It is proposed that the pro-inflammatory (M1) activation of microglia and macrophages includes two metabolic steps: early and late activation. (**A**) The early activation is mediated by P2X7Rs (**1**). Activated microglia relies on a glycolytic metabolism (**2**) followed by mitochondrial oxidation with an intact TCA cycle (**3**). A part of glucose is used in the pentose phosphate pathway (PPP) to obtain the substrates for biosynthesis and NADPH for the reduction of GSGG, gaining antioxidant power (**4**). (**B**) In the M1 late activation, microglia and macrophages overexpress P2X7Rs and GLUT (**1**). Despite the upregulation of Nrf2, excessive ROS are produced (**2**). ROS leads to mitochondrial impairment (**3**). TCA cycle has a deficient step in IDH and SDH with consequent accumulation of citrate and succinate (**4**), which favor the production of inflammatory cytokines. The brain microglial ROS may result in the formation of the NF-kB p50 radical, leading to the loss of function of Arg-1 and the consequent production of NO from L-arginine (**5**). NO drives the switch toward the aerobic glycolytic metabolism with the release of lactate. (**C**) M2 activation. The lactate in the ECM and the tumor-induced hypoxia stabilize the HIF1-α while the condition of glucose starvation constrains the cells to FAO metabolism (**1**) and mitochondrial rewiring. Glutamine-derived compounds enter the TCA cycle (anaplerosis) or are involved in the GSH synthesis (**2**). HIF-α and 2-HG restrain the inflammatory genes and promote anti-inflammatory behavior (**3**). Arg-1 upregulation reduces the substrate availability for the inducible nitric oxide synthase (iNOS) with consequent NO reduction (**4**). All the figures consider the metabolic changes and do not include the nuclear compartment. More detail in the text. ECM = extracellular matrix; ATP = adenosine triphosphate; P2X7Rs = purinergic receptor X7; MIP-1α = macrophage inflammatory protein 1-alpha; MCP1 = monocyte chemoattractant protein 1; glu = glucose; GLUT = Glucose Transporter; glu6P = glucose 6-phosphate; NADPH = nicotinamide adenine dinucleotide phosphate hydrogen; ROS = reactive oxygen species; GSGG = oxidized glutathione; GSH = Reduced glutathione; Ca^2+^ = calcium ion; I,II,III,IV,V = mitochondrial complex of phosphorylation electron chain; Q = coenzyme Q; C = cytochrome c oxidoreductase; TCA = tricarboxylic acid cycle; Nrf2 = Nuclear factor erythroid 2-related factor 2; Il-1β = Interleukin 1 beta; IDH = Isocitrate dehydrogenase; SDH = Succinate dehydrogenase; HIF-1 = Hypoxia-inducible factor 1; FAs = fatty acids; FFARs = free fatty acids receptor; αq-β-γ = G proteins coupled to receptor; FABP = fatty acid-binding protein; Arg-1 = Arginase-1; 2-HG = 2-hydroxyglutarate; NO = nitric oxide. Figure 1 was partially drawn using the Network Painter open-source tool from Stanford University.

During early glioma genesis, innate reactive microglia express inflammatory molecules [86,103]. Both murine and human pro-inflammatory microglia rely on glycolytic metabolism, accelerating the glucose turnover and oxidation, undergoing a structural mitochondrial remodeling for an energy-expensive event [75,104]. As suggested for other immune cells [105], it was proposed that the pro-inflammatory (M1) activation of microglia includes two metabolic steps [106], as an early activation can be distinguished from a late metabolic profile on the dependence of the tumor progression (Figure 1A,B). In the early phase, cells are able of utilizing both glycolytic and oxidative metabolism and activate the pentose phosphate pathway (PPP) for nucleotides synthesis and dihydronicotinamide-adenine dinucleotide phosphate (NADPH) generation. In the second stage, microglia switch to glycolytic metabolism for maintaining ATP levels, after the decrease of endogenous O_2_ consumption occurs. The decline of OXPHOS is due to the excessive ROS production induced by the inflammatory stimuli [107]. Brain microglial ROS may result in the generation of the NF-kB p50 radical, loss of NF-kB p50 function (DNA and protein binding interactions), leading to the accumulation of L-Arginine and increase of the pro-inflammatory response [108]. Hence, the inflammatory-related metabolic switch appears to be driven by inducible nitric oxide synthase (NOS) in dendritic cells and microglia, where nitric oxide (NO) is obtained from L-Arginine and NADPH [109,110,111]. The intracellular damage from ROS and reactive nitrogen species (RNS) is due to the generation of glutamate (taken up from the ECM or derived from a basal consumption of glutamine) and NADPH. Glutamate and NADPH are used for preventing the depletion of glutathione (GSH) and the activation of the nuclear factor erythroid 2-related factor 2 (Nrf2), which is involved in redox homeostasis and antioxidant responses in microglia [112]. In addition to enhanced glycolysis, M1 macrophages have an impaired TCA cycle with deficient steps at *IDH1* and succinate dehydrogenase (*SDH*), leading to citrate and succinate accumulation respectively, and damage in the mitochondrial respiration, since SDH is part of the mitochondrial electron transport system as complex II. Moreover, high levels of succinate promote the inflammatory response [113].

As GBM progresses, myeloid-derived cells undergo spatial transfer due to hypoxic area dependence and lactate production (Warburg effect). Tumor-induced hypoxia over time causes severe damage to endogenous cells in the immediate vicinity, edema formation at the margins, and necrosis in the core [114]. Microglia and macrophages in the area of the hypoxic niche are constrained to shift toward aerobic glycolysis [115] with the consequent increase in lactate production and lower acidic pH. The lactate stabilizes the expression of HIF-1 and promotes the M2 polarization of the macrophages, characterized by vascular endothelial growth factor (VEGF), arginase-1 (Arg-1), and low expression of major histocompatibility complex II (MHC II) molecules [116], therefore contributing to the immune-suppression mechanisms. Myeloid-specific deletion of the lactate dehydrogenases (LDH) in macrophages promotes the M1 phenotype with a consequent increase in the activation of CD3+ and CD8+ T lymphocytes-mediated anti-tumor immunity [117]. Arg-1 is known to metabolize L-arginine to ornithine and urea, limiting the supply of substrate to inducible NOS. Treatment with mammalian target of rapamycin (mTOR) inhibitor blocks the release of urea, thereby limiting the activity of Arg-1, impairing the M2 phenotype in microglia, and polarizing it toward the inflammatory profile [118,119]. This pathway may be involved in redirecting the metabolic state. Furthermore, in the microenvironment of a growing tumor, all elements of the CNS parenchyma compete for metabolic substrates, and glucose becomes scarce over time due to metabolic competition. M2 macrophages were shown to reduce glucose consumption and redirect metabolism towards fatty acid oxidation (FAO) and OXPHOS, as a mode of ATP production [106,120] (Figure 1C). Functional mitochondria should also be supplied by exogenous cells. Among the alternative substrates to glucose, these cells increase the consumption of glutamine [121]. The glutaminolysis provides intermediate compounds such as glutamate and α-KG. The α-KG may enter the TCA cycle for anaplerotic reactions, enhance FAO-OXPHOS activity, or restrain the inflammatory gene expression by preventing NF-kB activation while inducing a mitochondrial reprogramming via epigenetic regulation, supporting M2 polarization [122,123]. Downregulation of *inhibitor of nuclear Factor kappa B kinase subunit beta* (*IKK-β*) expression at the mRNA and protein level was found in M2 microglia and macrophages infiltrating human GBM [124]. As previously mentioned, mutations in the *IDH1*/*IDH2* genes are frequently observed in patients with secondary GBM. In addition to abolishing the production of α-KG, *IDH1*/*IDH2* mutations increase enzymatic activity and convert α-KG to the structurally similar molecule 2-hydroxyglutarate (2HG) [125]. Elevated 2HG levels in *IDH1/2*-mutated glioma are taken up by the stromal cells in the microenvironment. The oncometabolite 2HG impairs the inflammatory activation of microglia by inhibiting IKK activation, fine-tuning the immune response to GBM [126]. Collectively, evidence shows that NF-kB regulation is crucial in the remodeling of microglial functions exposed to GBM.

Indeed, the metabolic reprogramming of tumor-associated microglia and macrophages depends on specific microenvironment stimuli in tumor niches, since M1 macrophages are mainly found in oxygenated glioma regions and M2-polarized macrophages are increased in the hypoxic bulk core [127], further supporting the heterogeneity of the results in the literature and introducing the need of topographic studies. To be confirmed is whether M1 and M2 macrophages denote defined subpopulations rather than the shift between functional phenotypes on environmental cues [106]. In vitro results show a simultaneous glucose consumption, lactate release, and enhancement of mitochondrial production of energy in lipopolysaccharides (LPS)-stimulated-BV-2 microglia, a suitable alternative model of primary culture [111]. These data may suggest the existence of an alternative metabolic steady-state in which anaerobic fermentation and aerobic respiration occur at the same time.

## 4. GBM-Associated Astrocytes

### 4.1. Morphology

Astrocytes are native to the brain and could serve as cells of origin for GBM [128]. Like microglial cells and macrophages, they also perform immunological functions [129,130] and become activated with injury [131]. In the early stage of the malignancy, reactive astrocytes express the glial fibrillary acidic protein (GFAP+) and Nestin+ with a round shape body, often in mitosis, while after they retain the overexpression of GFAP, showing a larger cell size with thicker and longer processes [132]. Analogous, multiple subtypes may exist within murine and human GBM [133]. The immunolabeling for GFAP revealed both a gradual modification in the morphology—from fibrous to star shape—within the distance to the GBM, and an increasing density as the tumor grows [134]. Unlike the microglia, astrocytes are uniquely enriched in the peritumoral area [115] and form a sheet-like structure at the tumor edge [135]. Astrocytic cells are known to surround the sites of traumatic or toxic injury, forming a glial scar, providing the isolation of the damage, and a considerable impact on the repair or either a roadway for the injury expansion in the maladaptive interplay [136,137]. In the GBM samples, they usually surround the bulk, or less frequently are entrapped inside, appearing as gemistocytes [138], but physically connected with the tumor in a glial network. The growing, non-invasive GBM is surrounded by an astrogliosis capsule, while a dense network is found at the invasive edge, consequent to changes in the matrix composition [73]. This outcome may be explained by the CNS maladaptive plasticity beyond the ability of the GBM to modify the microenvironment for its benefits [101].

Astrocytes end-feet make contact with pericytes and closely entangle the endothelial cells for the proper maintenance of the blood–brain barrier (BBB), regulating the intra-brain influx of nutrients and the drug delivery [139]. Tumor cells “transform” the astrocytes enabling them to provide drug resistance and to favor growth and invasiveness [140,141,142,143]. Astrocytes and pericytes are disconnected in the perivascular space by cancer, giving the way for the tumor invasion. The structural coupling with the GBM (gap junctions) allows the astrocytes sequestration of calcium ions or the microRNAs transfer, preventing apoptosis induced by the chemotherapeutics [14,144,145]. Astrocytes and endothelial cell barrier confer protection from chemotherapeutics through the endothelin receptor signaling pathway in an orthotopic model of human GBM [146].

### 4.2. Molecular Profile

Astrocytic activation is driven by both the tumor and the activated innate immune system (microglia and macrophages) [115]. Evidence shows that microglial interleukin-6 (IL-6) signals the reactive astrocytes for reaching the peritumoral area and releasing Monocyte Chemotactic Protein-3 (MCP-3) via JAK-STAT proteins, attracting microglial cells, thus triggering a positive loop between microglia and astrocytes at the tumor site [147]. Transcriptome analysis of murine, purified, reactive astrocytes indicated the existence of an LPS-induced A1 subtype, and alternative A2 subtype [148]. The evidence suggests that the inflammatory A1-like reactivity is induced by activated microglia via pro-inflammatory cytokines and the extracellular release of fragmented mitochondria in neurodegenerative diseases [131,149]. GBM may contribute to the enrichment of the damaged mitochondria in the tumoral landscape, as GBM cells have a low number of mitochondria, thus suggesting high mitochondrial degradation activity [150] and early pro-inflammatory activation of astrocytes. In addition to the antigen presentation pathway, GBM-associated astrocytes show a gene signature that is distinguishable from those in lower-grade gliomas. Among these genes, *CD44* and *tenascin C (TNC)* were identified in the perivascular astrocytes [130].

At 6 days from the tumor injection, human astrocytes are defined by JAK/STAT pathway activation, an increased expression of CD274+, IL-10, and IFNγ secretion. This subtype of reactive astrocytes shows an anti-inflammatory state and significantly contributes to promoting tumor growth and invasion. Tumor-associated astrocytes mediate the transcriptional reprogramming in myeloid cells toward an anti-inflammatory phenotype and can directly inhibit the cytolytic lymphocytes by expressing PD-L1 and/or Fas ligand (FasL), thereby repressing anti-tumor immune functions [115]. Astrocytes overexpressing *STAT3* play a role in supporting radio resistance, scar formation, angiogenesis, and tumor invasion [151,152,153]. Reactive CD274^+^ astrocytes secrete the glycoprotein chitinase 3 like 1 (CHI3L1) for promoting a subtype-shift of glioblastoma towards the mesenchymal phenotype, driving mitogen-activated protein kinases (MAPK) signaling as well as high proliferation rate and migration [154]. GBM induces the neighbored astrocytes to release pro-MMP2 that is converted to MMP2 by GBM-derived plasminogen as well as by microglia-expressed MMP14. The tumor-derived astrocytes lead GBM cells to upregulate periostin and serglycin, which mediate the recruitment of M2 tumor-associated macrophages and mast cells, thereby enhancing its progression [60,143,155].

Different disease models showed many intermediate states between the A1 and the A2, which can be injury-, age-, and region-specific [59,148,156]. The use of new technologies like the single-cell RNA sequencing (scRNA-seq) conjugated with fluorescence-activated cell sorting (FACS) screening allowed the definition of heterogeneous subpopulations of astrocytes from different brain regions, with unique gene signatures, distinct molecular and functional properties, corresponding to their analogs in malignant glioma defined by distinctive genomic alterations [133]. Among these, the astrocyte subpopulation with enriched genes as *Rac2, Blcrb, Mrc2* and *Cd14*, serve malignancy and mostly overlap with the mesenchymal glioblastoma gene signature [157]. Indeed, the glioma heterogeneity and the immunomodulatory reshaping of the milieu operated by the GBM could be directly related to the astrocytes’ reactivity [158]. However, the molecular mechanisms in GBM cells and non-transformed, reactive astrocytes still have a differential regulation [159].

### 4.3. Metabolism

The recently described subpopulations of reactive astrocytes likely metabolize distinct energy substrates. In healthy conditions, astrocytes prefer a glucose-based metabolism and favor the production of lactate to meet the metabolic demand of the neurons [160], while largely employing mitochondrial OXPHOS for ATP production (called as Pasteur effect) [161]. Abundant mitochondria arranged in a fine network have been seen in their processes in vivo, supporting this function [162,163]. Reactive astrocytes dynamically modulate their metabolism, rapidly responding to environmental stress. However, how the metabolic dynamics in astrocytes are affected by GBM condition is largely unexplored [164]. Based on several pieces of evidence from tumor-derived astrocytes molecular patterns and other injury models, we suggested the astrocytic response as follows. In normoxic conditions, astrocytes’ metabolism is highly active, with a predominance of glycolysis and central carbon metabolites [165]. The first step is a transient increase in the glucose uptake through GLUT1 overexpression induced by peroxisome proliferator-activated receptor-gamma (PPARγ) and in the glucose oxidation via PPP, leading to the increase of the NADPH production, thus maintaining the reduction of GSH [166,167,168]. The uptake of glutamate increases and it is converted partially to α-KG, filling the TCA cycle. The mitochondrial respiratory chain rate is not affected in this phase, although a region-specific and transient reorganization of these organelles was proved in both LPS- and IFNγ-stimulated astrocytes with typical traits of gliosis [163,169]. The condition of glucose starvation due to the high metabolic demand of GBM cells may trigger glycogenolysis [170]. Paracrine energy and glycogen-related metabolites may be transferred by astrocytes to both the endothelial cells and the neurons, to maintain the BBB and the neurovascular unit [165].

The expression of PPARα may enhance FAO in the non-transformed, reactive astrocytes, still producing lactate as a source for feeding neurons [168,171]. The decrease in glucose uptake does not affect the mitochondrial ATP synthesis, which may increase according to a metabolic network modeling analysis [169]. Recently, a striking content in acylcarnitine produced during FAO was found at the GBM edge relative to the tumor core [172] and seemed essential for respiration and proliferation of glioma cells [173]. The active metabolism of the reactive astrocytes at the tumor edge and the pressure imposed by the growing GBM may cause the compression of the vessels, causing a malfunctioning of the brain endothelial barrier with perfusion-limited transient hypoxia at the tumor edge in the late stages of the tumor progression [135]. Under hypoxia, astrocytes maintain high glycolysis, amino acids, and nucleotide levels [165], and provide GBM cells of several nucleotides, particularly ATP, which are transferred through Cx43 [174] and secrete glutamine to replenish the TCA cycle in cancer cells [175]. The withdrawal of the glucose may impair the mitochondrial function with consequent excessive production of ROS [176] and a partial loss of mitochondria in the astrocyte processes [177]. Among the alternative sources of glucose, astrocytes may use glutamate or ketone bodies and produce energy via gluconeogenesis or ketolysis respectively during starvation [178,179,180]. Studies based on restriction calories and ketogenic diet show promising efficacy against the GBM [180,181], considering the normal cells as the only ones able to use the ketones [182,183]. The metabolic shift from glucose to ketone body metabolism promotes an anti-angiogenic, anti-inflammatory, and pro-apoptotic environment in the tumor mass [184]. The suppression of glycolysis and mitochondrial metabolism following the treatment with TG02, a pyrimidine-based multi-kinase inhibitor, induces cell death in glioblastoma cells but not in normal astrocytes. These preclinical data led to the launching of a phase I/II clinical trial (NCT02942264; [22]). However, these results may appear surprising if taking into account the high cancer cell metabolic plasticity, as discussed previously. The growth-promoting effects on U87 GBM of both ketone body and fatty acid supplementation under physiological glucose conditions were observed as well as hepatocellular carcinoma cells, which were shown to re-express the ketolytic enzyme succinyl-CoA: 3-ketoacid-CoA transferase (SCOT) during serum starvation [185,186]. These results suggested that further studies are needed to point out the metabolic response of both cancer and stromal cells, considering the phase of the disease.

## 5. GBM-Glial Cells Metabolism as Modules

Cancer cells coexist with host cells in several functioning states during the progression of the disease. Such heterogeneity in cell functioning must be targeted by therapies. The cell functional phenotype is managed by the bioenergetic machinery performance, which depends on the metabolism and regulates the gene expression. Metabolic changes are due to the interplay between cancer and the cells in the microenvironment and are accompanied by the modifications of oxygen and nutrients availability. Therefore, control of the metabolism both in cancer and host cells is a promising approach. However, metabolome analysis reveals that the metabolic network is complex and flexible. Complexity concerns hundreds of reactions occurring in different subcellular compartments at the same time; flexibility indicates that the same compound can be used in different metabolic chains.

Bioinformatic and computational studies help to disengage the complexity of the metabolism in modules [187]. Metabolic modules comprise a conserved sequence of chemical reactions for the transformation of a defined substrate. Metabolic modules present a comprehensive summary of the major metabolic activities and fulfill the production/usage of the main classes of metabolites (nucleotides, carbohydrates, lipids, and amino acids) [188].

**Figure 2:**
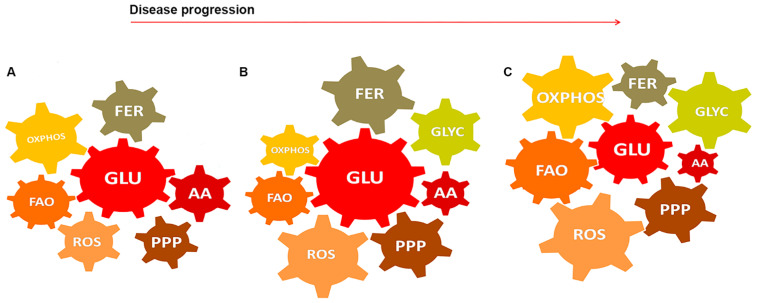
Metabolic modules in the GBM-glial cell network during the disease progression. (**A**) Metabolic modules may be considered as gears regulating the engine performance, that is the bioenergetic machinery in biological tissues. Based on the literature review, we hypothesized the relationships between the metabolic modules in the GBM-glial cells network. In physiological conditions, glucose oxidation-derived products allow both the OXPHOS, FER, and PPP. The ROS production is counterbalanced by the AA pool, which is partially used for synthesizing anti-oxidant agents. Fatty acid oxidation also precedes ATP production and substrates for biosynthesis. (**B**) With the presence of GBM, metabolic and mitochondrial rewiring occurs. Both cancer and non-cancer cells are supposed to rely on glycolytic metabolism and PPP for ATP and biosynthesis need respectively. ROS production exceeds and impairs the mitochondrial OXPHOS. Astrocytes maintain the FER to fulfill the neurons’ metabolic needs. Glycogen and non-essential AA are recruited as an alternative source of glucose. (**C**) In the condition of glucose starvation, glycogen deposits and AA keep on fuel the energetic and biosynthetic metabolism [189]. FAO acquires a major role to sustain the OXPHOS, but the overproduction of ROS may trigger new adaptations and maladaptive plasticity. Glucose oxidation (glycolysis, GLU); anaerobic fermentation (FER), amino acids (AA), oxidative phosphorylation (OXPHOS); reactive oxygen species (ROS); pentose phosphate pathway (PPP); fatty acid oxidation (FAO); glycogenolysis (GLYC).

Considering the metabolic changes reviewed here due to GBM and glial cells interaction during the disease progression, we could identify eight main metabolic modules: glucose oxidation (glycolysis, GLU), anaerobic fermentation and lactate production (FER), PPP, (non-essential) amino acid pool (AA) such as glutamate, OXPHOS, FAO, ROS production, glycogenolysis (GLYC) (Figure 2). Each module could be analyzed separately in 3D models of GBM-neuro/immune interactions for every phase of the disease, and these fluxes could be integrated to form a feasible flux in the whole network. These may help to identify the key factors for controlling the metabolism in targeted, time-dependent therapies and become tractable for large-scale networks. Morphological changes and the molecular profiles in the whole network could be investigated with the same approach and used for correlative analysis, gaining insights in understanding the mechanisms underlining the studied functional phenotypes.

## 6. Conclusions

There is a paucity of data concerning the role of innate immune cells in GBM maintenance, and standard glioma therapies do not account for the effects of the glial cells within the tumor microenvironment. Indeed, this may be one possible reason for the lack of improvements for patients with high-grade gliomas therapies. The cell morphology serves several functions that are consequent to the bioenergetic machinery fitness. The cell metabolism depends, in turn, on the availability of nutrients and interactions in the microenvironment, which varies depending on the location in the tumor mass and proximity to blood vessels, underlying the impact of the context in anti-tumor therapeutic approaches.

Single-cell profiling obtained combining gene expression, sequencing data, and protein levels reveals the complexity and the heterogeneity of immune cell functioning states related to GBMs. Variation in OXPHOS rate and regulation appears to be the most important contributor to the metabolic and functional heterogeneity among malignant and non-malignant cells. Of note, OXPHOS activity is correlated with both glycolysis and response to hypoxia in almost all cell types and might be responsible for adapting to environmental factors [190]. Thus, refined strategies aiming at modulating the mitochondrial functions in the selected cell populations will have to be combined and tested for therapeutic potential to give relevance to the discovery of metabolic modules controlling GBM progression and providing valuable insights into the specificity of the immune response [191,192].

Systems biology provides dynamic challenges and consequent adaptation over time. The metabolic and functional features of the cells vary during the tumor crosstalk and across the portion of the GBM, thereby underlying the necessity to identify metabolic modules as a function of time and gradient. Drugs administered against the GBM may fail because of the pH and molecular composition changes across the tumor bulk [172], as well as because of the evolution of the tumor and its microenvironment. As a result, time-related evidence was reported on the outcome of standard treatments used to treat patients with GBM [193,194,195].
